# The Monkey Solves the Problem

**Published:** 1891-09

**Authors:** 


### The Monkey Solves the Problem.

Monkeys have a keen sense of imitation and are always prone to copy there masters movements whenever fancy strikes them. Seldom, however, is it that a monkey has proved itself useful by such an undesirable propensity. Yet one of these inquisitive creatures has, we understand, recently performed a feat in the matter of medecine-taking, and by so doing has earned for itself a reputation which deserves recognition. This is how it was: A practitioner recently received a box of Count Mattei's medicines, and one of his children getting hold of the box gave it to a tame monkey in the house. The animal very soon broke open the box, and taking a vial of anticanceroso, which is used as a cure for leprosy, swallowed 750 globules, besides some other fever medicines. The proper method of taking the anti-canceroso is to dissolve one of the globules in a quart of water, and the dose is a teaspoonful at a time. The monkey, however, is not only quite well, but as lively as ever, and must now be impervious to leprosy. Clearly, if the monkey had been able to read he would have been more discreet with Count Mattei's remedies. but as no harm happened to him, the presumption is that the remedies, are harmless however they are taken.—*Medical Press.*

